# Refractive Status in Eyes Implanted with Toric and Nontoric Intraocular Lenses during Combined Cataract Surgery and Microhook Ab Interno Trabeculotomy

**DOI:** 10.1155/2021/5545007

**Published:** 2021-05-29

**Authors:** Yasuyuki Takai, Kazunobu Sugihara, Mihoko Mochiji, Kaoru Manabe, Aika Tsutsui, Masaki Tanito

**Affiliations:** Department of Ophthalmology, Shimane University Faculty of Medicine, Izumo 693-8501, Japan

## Abstract

**Purpose:**

To compare the refractive status between eyes implanted with toric and nontoric intraocular lenses (IOLs) during combined cataract surgery and microhook ab interno trabeculotomy (*μ*LOT), a minimally invasive glaucoma surgery (MIGS).

**Methods:**

Twenty eyes of 20 patients who had open-angle glaucoma, cataract, and preexisting regular corneal astigmatism exceeding 1.5 diopters (D) and underwent combined *μ*LOT and phacoemulsification were recruited retrospectively. Ten eyes were implanted with a toric IOL and 10 eyes with a nontoric IOL. The primary outcomes were the uncorrected visual acuity (UCVA) and refractive cylinder at 3 months postoperatively.

**Results:**

The mean UCVA of the toric IOL group (logarithm of the minimum angle of resolution (logMAR), 0.23 ± 0.25) was significantly better than that of the nontoric IOL group (logMAR, 0.45 ± 0.26) at 3 months postoperatively (*p* < 0.05). The mean absolute residual refractive cylinder of the nontoric IOL group (2.25 ± 0.62 D) was significantly greater than that of the toric IOL group (1.30 ± 0.68 D) (*p* < 0.05). Postoperatively, 60% of eyes in the toric IOL group and 10% in the nontoric IOL group had an absolute refractive astigmatism level of 1.5 D or less. Surgically induced astigmatism (0.77 ± 0.43 D for toric group and 0.60 ± 0.32 D for nontoric group) and IOP reduction (33.9 ± 15.6% for toric group and 29.4 ± 11.7% for nontoric group) were not different between groups.

**Conclusions:**

Use of toric IOL during combined cataract surgery and *μ*LOT is possible and better than not, but physician should prevent their patient of persisting residual astigmatism. The study was registered at https://www.umin.ac.jp/, and the clinical trial accession number is https://clinicaltrials.gov/ct2/show/UMIN000043141.

## 1. Introduction

**Figure 1:**
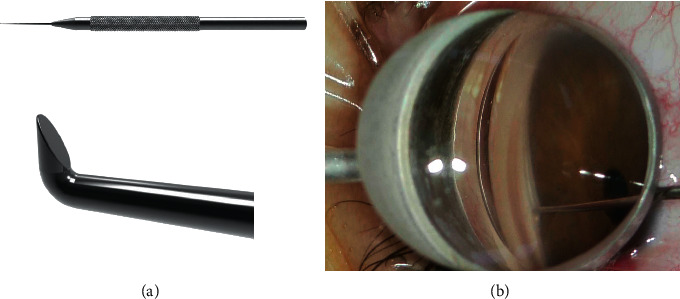
Microhook and intraoperative findings of microhook ab interno trabeculotomy. (a) The image shows the spatula-shaped microhook designed for use during ab interno microhook trabeculotomy (M-2215, Inami, Tokyo, Japan) and a photomacrograph of the tip of the microhook. (b) The tip of the microhook is inserted directly into Schlemm's canal temporally and moved circumferentially to incise the inner wall of Schlemm's canal and trabecular meshwork.

Glaucoma is the leading cause of irreversible blindness globally. Glaucoma surgery is necessary when maximally tolerated medical therapy or laser treatments cannot control disease progression. Trabeculectomy (LEC) remains the standard surgical procedure for glaucoma to reduce the intraocular pressure (IOP). The astigmatic changes after LEC can lead to decreased visual acuity (VA) and might be problematic for patients [1]. As a result, a number of investigators have studied corneal refractive changes or surgically induced astigmatism (SIA) resulting from LEC [2, 3]. Minimally invasive glaucoma surgeries (MIGS) have been developed as safer and less invasive procedures with moderate IOP reduction and earlier treatment options compared with traditional surgery; these procedures often are performed in combination with cataract surgery. Despite the popularity of MIGS procedures on IOP reduction and safety, the impact of glaucoma surgical techniques on refraction, particularly astigmatism, is incompletely understood. While visual changes can result from decreased IOP [4], the direct effect of glaucoma surgery on the corneal topography is not fully known [2]. Microhook ab interno trabeculotomy (*μ*LOT) is a novel procedure that uses microhooks and is a Schlemm's canal MIGS procedure reported by Tanito et al. (Figures 1(a) and 1(b)) [5]. The degrees of SIA were calculated to be 1.01, 0.62, 0.23, and 0.12 for LEC, EX-PRESS® shunt (Alcon Vision LLC, Fort Worth, TX, USA), ab externo trabeculotomy, and *μ*LOT groups, respectively [6]. Accordingly, minimal induction of astigmatism and less astigmatism-related decreases in the BCVA would be expected with the *μ*LOT procedure. Recently, toric IOLs have been implanted increasingly in patients with corneal astigmatism during both cataract surgery alone and combined microincision vitrectomy surgery and cataract surgery to achieve postoperative spectacle-free distance vision [7, 8]; however, the use of toric IOL during MIGS is limited and far between in the literature [9], and the correcting astigmatism effect of toric IOL during combined MIGS is unclear.

AcrySof® IQ Toric IOL (Alcon Vision LLC, Fort Worth, TX, USA) had good rotational stability and favorable efficacy in patients with cataracts and corneal astigmatism [10]. To our knowledge, AcrySof® IQ Toric IOL implantation combined *μ*LOT in patients who had cataract and preexisting astigmatism, and glaucoma has never been reported previously. The purpose of this study was to investigate the refractive changes in eyes implanted with AcrySof toric IOLs compared with nontoric IOLs combined with *μ*LOT to treat glaucoma with preexisting corneal astigmatism.

## 2. Methods

The Ethics Committee of Shimane University Hospital approved the current study, which adhered to the tenets of the Declaration of Helsinki. The ethics committee waived the requirement for patients' informed consent regarding the use of their medical record data in accordance with the regulations of the Ethical Guidelines for Medical and Health Research Involving Human Subjects issued by the Japanese Government, and, instead, the protocol was posted on the department homepage to notify the potential participants about the study. We reviewed retrospectively the medical records of Japanese eyes, i.e., 10 consecutive eyes with open-angle glaucoma (OAG) implanted with a toric IOL and 10 eyes implanted with a nontoric IOL of patients who had preoperative regular corneal astigmatism exceeding 1.5 diopter (D) and underwent combined phacoemulsification and *μ*LOT at Shimane University Hospital from February 2016 to December 2018. In our department, we began to use toric IOLs during combined *μ*LOT procedure after 2018; therefore, most eyes in the nontoric group underwent surgery earlier in the study period, and those in the toric groups underwent surgery later in the study period. All patients underwent thorough ophthalmologic examinations that included measurements of the uncorrected VA (UCVA) and best-corrected VA (BCVA) values using a Landolt decimal acuity chart, refraction, and axial length measured by OA2000 (Tomey, Nagoya, Japan), number of glaucoma medications, and indirect ophthalmoscopy; the VA measurements were converted into the logarithm of the minimal angle of resolution (logMAR) VA. The inclusion criteria included OAG with cataract without any other ocular disease. Eyes were included that had no history of a previous intraocular surgery, no central visual field defect due to glaucoma, no complications during combined *μ*LOT and cataract surgery, and no postoperative interventions. Patients who met those criteria and were implanted with an AcrySof ® IQ Toric IOL (Alcon Vision LLC, Fort Worth, TX, USA) were assigned to the toric IOL group; patients implanted with a nontoric IOL (XY-1, HOYA Corp., Tokyo, Japan) were assigned to the nontoric IOL group. The IOL spherical power was calculated for each case using the Barrett formula and the targeted refraction was emmetropia. In the toric IOL group, the IOL cylinder power and alignment axis were calculated using a web-based toric IOL calculator (available at http://www.acrysoftoriccalculator.com/) considering the keratometry readings and mandatory data input on the position of the incision and SIA by a superior incision (0.10 D). The manufacturer's information indicated that the SN6AT3, SN6AT4, SN6AT5, SN6AT6, SN6AT7, SN6AT8, and SN6AT9 toric IOLs (Alcon Vision LLC, Fort Worth, TX, USA) are intended for use when preexisting corneal astigmatism levels are about 1.03, 1.55, 2.06, 2.50, 3.00, 3.50, and 4.00 D, respectively.

### 2.1. Sample Size

A previous study that compared the absolute residual refractive astigmatism after cataract surgery between patients with cataracts and preexisting corneal astigmatism implanted with the AcrySof® IQ Toric IOL and AcrySof® spherical control IOL reported that the mean absolute residual refractive cylinder was 0.59 D after toric IOL implantation versus 1.22 D after nontoric IOL implantation [11]. If the difference in the residual refractive cylinder between the two groups was 0.33 D [12] with a significance level of 0.05 and a power of 0.8, an estimated sample size of at least six eyes in each group was calculated as essential for detecting a significant difference between the two groups. Considering the possibility of increasing the standard deviation, 10 eyes were enrolled in each group (a total of 20 eyes).

### 2.2. Surgical Method

Two surgeons (YT and MT) performed all surgeries. In the toric IOL group, a preoperative toric reference corneal marker (#17251 Moria S.A., Antony, France) was used to place two limbal reference marks at the 9 and 3 o'clock positions with the patient sitting upright. Cataract extraction was performed similarly in both groups through 2.4 mm superior limbal incisions. In the toric IOL group, the actual implantation axis was marked using an intraoperative toric axis marker (#17250, Moria S.A), and the toric IOL was implanted using a Monarch III injector (Alcon Vision LLC, Fort Worth, TX, USA). *μ*LOT was performed through two corneal side ports as reported previously [13]. Briefly, a spatula-shaped microhook designed specifically for use during *μ*LOT was used (M-2215, Inami, Tokyo, Japan) (Figure 1(a)). An ophthalmic viscosurgical device (PROVISC, Alcon Vision LLC, Fort Worth, TX, USA) was injected into the anterior chamber through two corneal side ports. A microhook was inserted into the anterior chamber through the corneal port using a Swan-Jacob gonioprism lens (Ocular Instruments, Bellevue, WA, USA) to observe the angle opposite to the corneal port. The tip of the microhook then was inserted into Schlemm's canal and moved circumferentially to incise the inner wall of Schlemm's canal and trabecular meshwork over 3 clock hours (Figure 1(b)). Using the same procedure, *μ*LOT was performed in the opposite angle. After removal of the viscoelastic material, the IOL was rotated to align the cylinder axis to the marked axis. At the end of surgery, 2 mg of betamethasone sodium phosphate (Rinderon, Shionogi Inc., Osaka, Japan) was injected subconjunctivally and 0.3% ofloxacin ointment (Tarivid, Santen Pharmaceutical, Osaka, Japan) was applied. Postoperatively, 1.5% levofloxacin (Pfizer Japan Inc., Tokyo, Japan) and 0.1% betamethasone (Sanbetason, Santen Pharmaceutical) were applied topically four times daily for 4 weeks in all cases.

### 2.3. Primary Outcomes

The primary outcomes were the UCVA and absolute residual refractive cylinder at 3 months postoperatively. The astigmatic outcomes were calculated and visualized using the American Society of Cataract and Refractive Surgery Astigmatism Double Angle Plot Tool version 1.1.0 based on the vector analysis algorithm. With this tool, the preoperative corneal astigmatism was compared to the postoperative refractive astigmatism. The SIA was evaluated using SIA Calculator Tool (available at https://www.doctor-hill.com/iol-main/toric_sia_calculator.htm).

### 2.4. Statistical Analysis

Statistical analyses were performed using JMP version 11 (JMP Statistical Discovery, Cary, NC, USA). The differences between the preoperative and postoperative UCVA, BCVA, and refractive astigmatism were compared using the Wilcoxon signed-rank test. The differences between two groups were compared using the Mann-Whitney *U* test. For all statistical tests, *p* < 0.05 was considered significant.

## 3. Results

The demographic and clinical data from the toric and nontoric groups are summarized in Table 1. No significant differences were seen between the two groups in age, preoperative BCVA, absolute refractive cylinder, IOP, and number of glaucoma medications. The mean calculated target postoperative residual astigmatism was 0.57 ± 0.20 D. One eye (10%) each was implanted with SN6AT3, SN6AT4, and SN6AT7 IOLs, three eyes (30%) were implanted with SN6AT5 IOL, and four eyes (40%) were implanted with SN6AT6 IOL. No intergroup differences were seen in the BCVA and IOP preoperatively.

The status of the VA, astigmatism, and IOP at 3 months postoperatively is shown in Table 2. No intergroup difference was seen in the BCVA, while the UCVA in the toric IOL group was significantly better than that in the nontoric IOL group. The mean absolute residual refractive cylinder in the toric IOL group (1.30 ± 0.68 D) was significantly smaller than that in the nontoric IOL group (2.25 ± 0.62 D). No significant differences were seen in the corneal SIA, IOP, number of glaucoma medications, and reduction of IOP between the two groups.

**Figure 2:**
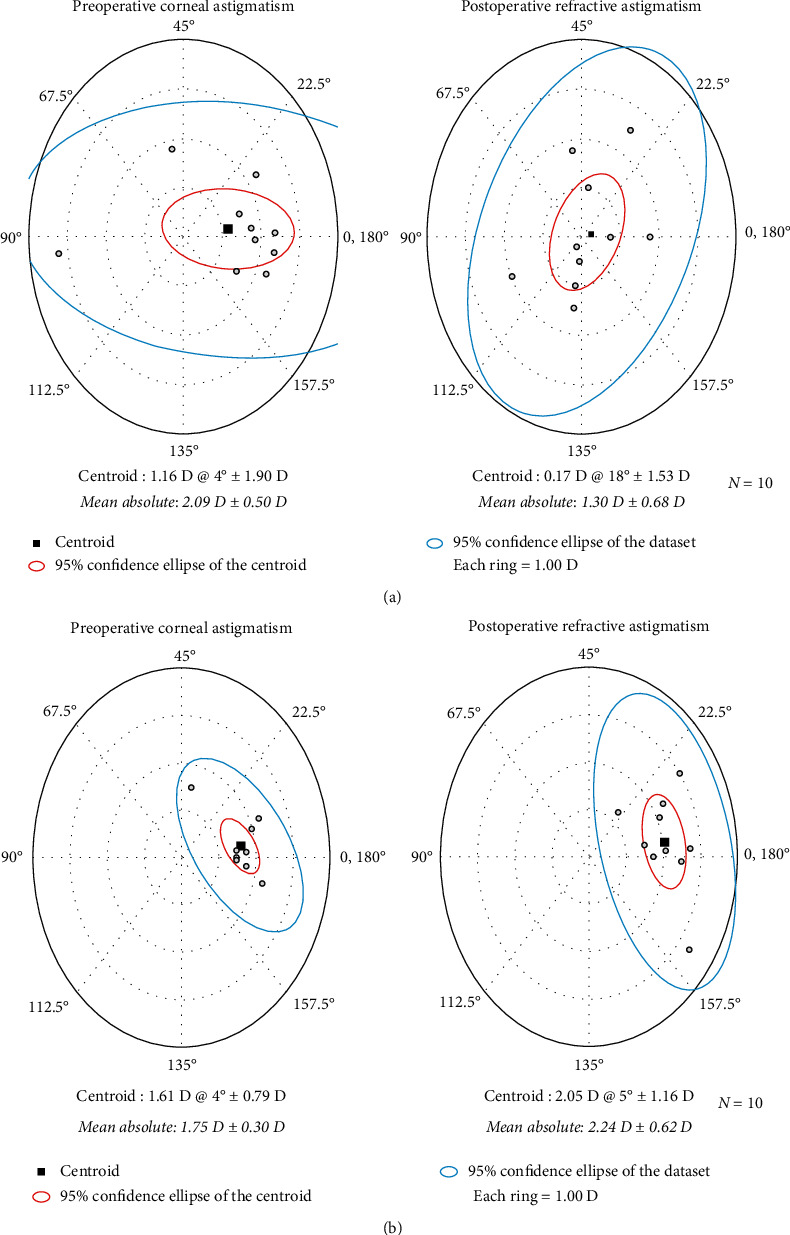
The double-angle plots show the preoperative and postoperative astigmatic vectors and their means and spread in the toric IOL (a) and nontoric IOL groups (b). The black squares indicate the centroids, the red circles indicate the 95% confidence ellipses of the centroids, and the blue circles indicate the 95% confidence ellipses of the dataset.

**Figure 3:**
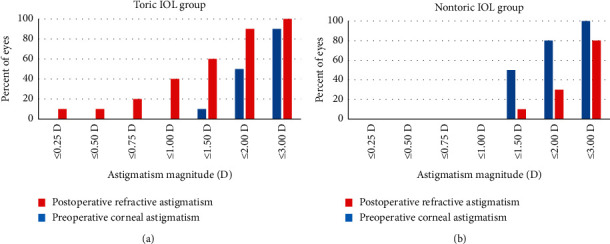
The magnitude of the preoperative corneal astigmatism and postoperative refractive astigmatism in the toric IOL and nontoric IOL groups.

Preoperative and postoperative astigmatic vectors and their means and distributions are shown in the double-angle plots in Figure 2(a) and 2(b) for the toric and nontoric IOL groups, respectively. Preoperatively, the centroids of corneal astigmatism in the toric (1.16 ± 1.90 D at 4 degrees) and nontoric (1.61 D ± 0.79 D at 4 degrees) groups were similar, while the centroids of postoperative residual astigmatism in the toric group (0.17 D ± 1.53 D at 18 degrees) were much smaller than those in the nontoric group (2.05 D ± 1.16 D at 5 degrees). The percentage of eyes with an absolute refractive astigmatism of 1.5 D or less was 60% in the toric IOL group and 10% in the nontoric IOL group (Figure 3).

## 4. Discussion

In the current study, postoperative UCVA was significantly better in toric IOL group than in nontoric IOL group due to the less refractive astigmatism, clearly presenting the efficacy of toric IOL use during combined cataract surgery and *μ*LOT for correction of preexisting regular corneal astigmatism. This observation is unique in the literature.

LEC is the most commonly performed glaucoma surgery. Substantial evidence indicates that LEC is associated with significant astigmatic changes and increased refractive surprises, suggesting that more invasive glaucoma surgeries are not refractively neutral [2]. The possible mechanism of SIA after LEC may be tissue contraction around the LEC site secondary to extensive scleral cautery and suture, removal of the second scleral flap, the wound-healing process of the subconjunctiva [3, 14], and corneal steepening provoked by the pressure of a large drainage bleb under the eyelid [15]. In addition, Delbeke et al. reported that the lower IOPs achieved after filtration surgeries were associated with higher SIA and worse VA [4]. As the unpredictable astigmatic changes after LEC, toric IOL had been hardly used to correct preexisting regular corneal astigmatism so far. MIGS were developed to achieve safer and less invasive interventional treatments earlier in the disease process and are useful for moderately reducing IOP and/or medication dependence in combination with already planned cataract surgery to address visual disturbances associated with glaucoma. Therefore, patients treated with MIGS may place more importance on good visual quality than on IOP reduction because of their anosognosia with visual field loss. In the current study, combined *μ*LOT and phacoemulsification with toric IOL implantation is an effective method to achieve better UCVA by correcting preexisting corneal astigmatism. In *μ*LOT, conjunctival and scleral sparing with the ab interno technique, short surgical time, moderate IOP reduction, and no bleb-related complications [13] may contribute to the minimal induction of astigmatism and less astigmatism-related decreases in the BCVA. The SIA after combined *μ*LOT and cataract surgery in the current study (0.77 ± 0.43 D in the toric IOL group and 0.60 ± 0.32 D in the nontoric IOL group) did not differ greatly from that after microincisional cataract surgery (mean SIA magnitude, 0.42 D after a 1.8 mm incision coaxial phacoemulsification and 0.5 D after 1.7 mm incision bimanual phacoemulsification) [16]. Manoharan et al. reported that refractive surprises occurred more often in patients with glaucoma, particularly those with angle-closure and pseudoexfoliation glaucoma treated with cataract surgery alone [17]. In two patients of the toric IOL group with high myopia and pseudoexfoliation (PXF), slight deterioration of postoperative absolute refractive astigmatism compared to the preoperative values was observed. Postoperative refractive astigmatism is thought to include toric IOL rotation, lens capsule (and/or IOL) tilt, decentration, and unknown ocular components in addition to corneal astigmatism [18]. In the high myopic patients with lax capsule and PXF patients with zonular weakness, IOL and capsule tend to rotate and tilt [18, 19]. In the current study, postoperative toric IOL rotation was not examined, and IOL rotation and/or lens capsule IOL tilt after surgery may have occurred in our two cases; thus, we should take notice of the possibility to not correct the preoperative astigmatism in the patients with high myopia and PXF. Saheb and Ahmed reported that, compared to patients with OAG who underwent phacoemulsification alone, combined cataract and endoscopic cyclophotocoagulation (ECP) surgery had more myopic outcomes than predicted refractive outcomes [20]. Those authors hypothesized that because ECP targets the ciliary body, which is connected to the lens zonules, ciliary body changes resulting from ECP had the potential for unpredictable refractive outcomes. However, previous studies have reported that the combined trabecular microbypass, trabectome, and phacoemulsification procedures had similar refractive outcome to cataract surgery alone [21, 22]. They suggested that permanent removal of a portion of the trabecular meshwork and increased aqueous outflow did not result in significantly different refractive outcomes compared to cataract surgery alone. Thus, the subtypes of glaucoma and/or kinds of MIGS may affect the refractive outcomes, but this remains unclear. In the current study, less refractive surprises occurred; therefore, *μ*LOT is not a factor that affects the final SIA when treating preexisting corneal astigmatism with glaucoma as previously described [6]. The strength of current results also suggested that AcrySof® IQ Toric IOL implantation in patients treated with combined *μ*LOT and phacoemulsification is a favorable option for correcting preexisting corneal astigmatism and achieving better UCVA. In addition, 79% of patients treated with µLOT achieved successful IOP control of 18 mmHg or less and a 15% reduction or greater [13]; while the surgical indication for *μ*LOT is for mild-to-moderate glaucoma, most patients treated with combined *μ*LOT and phacoemulsification with toric IOL implantation more likely will achieve good visual quality, that is, good UCVA for years until the next surgical intervention such as LEC or tube-shunt surgery. With the unpredictable astigmatic changes after LEC, toric IOL had been hardly used to correct preexisting regular corneal astigmatism so far, then the weakness of toric IOL use in *µ*LOT may have a negative influence on VA as an increase in aberration when the patients undergo LEC in the future. Thus, the further long-time observational studies are necessary to confirm the efficacy and safety. The IOP reduction (33.9 ± 15.6% for toric group and 29.4 ± 11.7% for nontoric group) was not different between groups and the same as previous reports in combined *µ*LOT and phacoemulsification [5]; this suggested that use of toric IOL did not affect the treatment effect.

## 5. Conclusions

Combined *μ*LOT and phacoemulsification with AcrySof® IQ Toric IOL implantation is possible and better than not but physician should prevent their patient of persisting residual astigmatism. The current study was limited by its retrospective nature and short follow-up period. Further randomized and prospective studies may confirm the efficacy and safety of the combined *μ*LOT and phacoemulsification with toric IOL implantation, and our study can be a useful reference for future trials.

## Figures and Tables

**Table 1:** Demographic and preoperative clinical data.

	Toric IOL group	Nontoric IOL group	*p* value
No. of eyes (patients)	10 (10)	10 (10)	
Mean age (years)	74.6 ± 7.20	76.9 ± 6.82	0.5943^a^
Gender (male/female)	3/7	6/4	0.1775^b^
Mean preoperative BCVA (logMAR)	0.19 ± 0.23	0.26 ± 0.4	0.8194^a^
Mean absolute preoperative corneal cylinder (D)	2.09 ± 0.50	1.75 ± 0.30	0.0495^a^
Median absolute preoperative corneal cylinder (D)	2.05	1.63	
(Minimum—maximum)	1.52–3.25	1.50–2.25	
Mean absolute preoperative refractive cylinder (D)	2.70 ± 0.90	2.08 ± 0.58	0.1147^a^
Median absolute preoperative refractive cylinder (D)	2.75	2.13	
(Minimum—maximum)	1.25–4.00	1.51–3.00	
IOP (mmHg)	19.5 ± 6.17	18.8 ± 3.55	0.8197^a^
Medication	2.70 ± 1.06	2.70 ± 0.80	0.4453^a^
*Toric IOL, no. (%)*			
SN6AT3	1 (10)		
SN6AT4	1 (10)		
SN6AT5	3 (30)		
SN6AT6	4 (40)		
SN6AT7	1 (10)		

BCVA: best-corrected visual acuity; log MAR: logarithm of the minimum angle of resolution; IOP: intraocular pressure; IOL: intraocular lens. ^a^Wilcoxon signed-rank test. ^b^Mann–Whitney *U* test.

**Table 2:** Postoperative visual acuity, astigmatism, and IOP at 3 months in the toric IOL and the nontoric IOL groups.

	Toric IOL group	Nontoric IOL group	*p* value
UCVA (logMAR)	0.23 ± 0.25	0.45 ± 0.26	0.0430^∗^
BCVA (logMAR)	−0.11 ± 0.08	0.05 ± 0.12	0.2528
Mean absolute postoperative refractive cylinder (D)	1.30 ± 0.68	2.25 ± 0.62	0.0111^∗^
Median absolute postoperative refractive cylinder (D)	1.25	2.25	
(Minimum—maximum)	0.25–2.50	1.25–3.25	
SIA (D)	0.77 ± 0.43	0.60 ± 0.32	0.3445
IOP (mmHg)	12.4 ± 3.41	13.0 ± 1.70	0.5421
Medication	1.70 ± 0.95	2.10 ± 0.88	0.3069
IOP reduction (%)	33.9 ± 15.6	29.4 ± 11.7	0.5706

UCVA: uncorrected visual acuity. The *p* values are calculated by Wilcoxon signed-rank test. ^∗^Significance levels of 5%.

## Data Availability

The data that support the findings of this study are available from the corresponding author.
